# Adipokines in pulmonary hypertension: angels or demons?

**DOI:** 10.1016/j.heliyon.2023.e22482

**Published:** 2023-11-17

**Authors:** Qi Jia, Yeling Ouyang, Yiyi Yang, Shanglong Yao, Xiangdong Chen, Zhiqiang Hu

**Affiliations:** a Department of Anesthesiology, Union Hospital, Tongji Medical College, Huazhong University of Science and Technology, Wuhan, 430022, China; b Institute of Anesthesia and Critical Care Medicine, Union Hospital, Tongji Medical College, Huazhong University of Science and Technology, Wuhan, 430022, China; c Key Laboratory of Anesthesiology and Resuscitation (Huazhong University of Science and Technology), Ministry of Education, China

**Keywords:** Pulmonary hypertension, Obesity, Adipokines, Vascular remodeling, Leptin, Adiponectin

## Abstract

Pulmonary hypertension (PH) is a devastating cardiopulmonary disorder with poor prognosis and limited curative options. Recent studies revealed a strong association between adipose tissue dysfunction (e.g., obesity) and PH. Adipokines are bioactive polypeptides with pleiotropic effects mainly produced by adipose tissue, and it is suggested that imbalanced production of adipokines in obesity may play a key role in the pathogenesis of PH. Alternations in the production and secretion of adipokines have been observed in PH patients and rodents PH models. In this review, we summarize the expressions and functions of several well-recognized adipokines, the roles of adipokines in the pathogenesis of PH and recent advances in the pharmacological and molecular modulation of adipokines in the treatment of PH. We found that several adipokines (e.g., leptin, resistin, and chemerin) have been demonstrated to display pro-proliferation, pro-inflammatory, and pro-oxidative properties and exacerbate PH. Other adipokines (e.g., adiponectin, apelin, and omentin-1) have anti-proliferation, anti-inflammatory, anti-fibrotic and anti-oxidative impacts on the pulmonary vascular remodeling of PH and are suggested as protective factors against PH, and targeting imbalanced adipokines appears to be a potential novel therapeutic strategy for the treatment of PH.

## 1 Introduction

Pulmonary hypertension (PH) is a lethal and multifactorial cardiopulmonary disease characterized by progressive obstructive remodeling of resistance pulmonary arteries, leading to right ventricular failure and premature death [1]. Based on the causes, PH can be divided into 5 different groups (Table 1) [1]. Although multiple targets and signaling pathways, including transforming growth factor β (TGF-β)/bone morphogenetic protein (BMP) signaling, hypoxia-induced Factor (HIF) signaling, vascular endothelial growth factors (VEGF) and endothelial-1 (ET-1), have been demonstrated to be involved in the pathogenesis of PH [[2], [3], [4]], however, no efficient curative therapy has been developed to reverse the progression of PH. Therefore, a better understanding of PH pathogenesis is urgently needed to develop more efficient curative treatment for PH patients.

**Table 1:** Updated classification of pulmonary hypertension (PH).

Group 1-Pulmonary arterial hypertension (PAH)
1.1 Idiopathic PAH
1.2 Heritable PAH (e.g., *BMPR2, ALK1 and ENG* mutation)
1.3 Drug- and toxin-induced pah
1.4 PAH-associated with (1.4.1 Connective tissue disease; 1.4.2 HIV infection; 1.4.3 Portal hypertension; 1.4.4 Congenital heart diseases; 1.4.5 Schistosomiasis)
1.5 PAH long-term responders to calcium channel blockers
1.6 PAH with overt features of venous/capillary involvement (PVOD/PCH)
1.7 Persistent PH of the newborn syndrome
**Group 2-PH due to left heart disease**
2.1 Left ventricular systolic dysfunction
2.2 Left ventricular diastolic dysfunction
2.3 Valvular heart disease
2.4 Congenital/acquired left heart inflow/outflow tract obstruction and congenital cardiomyopathies
**Group3- PH due to lung diseases**
3.1 Chronic obstructive pulmonary disease
3.2 Interstitial lung disease
3.3 Other pulmonary diseases with mixed restrictive and obstructive pattern
3.4 Sleep disordered breathing
3.5 Alveolar hypoventilation disorders
3.6 Chronic exposure to high altitude
3.7 Developmental lung diseases
**Group 4- PH due to chronic thromboembolism**
**Group 5- PH due to other causes**
5.1 Haematological disorders
5.2 Systemic and metabolic disorders
5.3 Others
5.4 Complex congenital heart disease

BMPR2: bone morphogenic protein receptor type II; ALK1: activin receptor-like kinase 1; ENG: endoglin (referenced by Ref. [1]).

Recently, mounting evidence revealed a strong association between obesity and PH [5,6]. Obesity is a well-recognized independent cardiovascular risk factor and a comorbidity to PH, and 30–40 % of patients with PH are reported to be obese [7]. In PH patients with morbid obesity, significant hemodynamic improvements in pulmonary circulation can be achieved after bariatric surgery [8,9]. In obesity, the excess accumulation of body fat leads to systemic low-grade inflammation, insulin resistance, and oxidative stress that may also affect the pathogenesis of PH [6]. In addition, obesity mediated-adipose tissue dysfunction is a key mechanism that may contribute to the development of PH [8,10,11]. For example, in obesity, adipose tissue dysfunction results in a marked changes of oestrogen synthesis and metabolism (e.g., increased production of urinary 16α-hydroxyestrone and cytochrome P450 1B1), which promotes pulmonary artery smooth muscle cell (PASMC) proliferation, increases oxidative stress, and exacerbates PH [6]. Furthermore, obesity mediated-adipose tissue dysfunction also led to alternation of other bioactive mediators (e.g., adipokines) production and secretion, which may also involve in the pathogenesis of PH [10].

Adipose tissue is increasingly recognized as an important endocrine organ that is implicated in regulating vascular and metabolic homeostasis as well as in the inflammatory response through the production of various bioactive polypeptides termed adipokines [12,13]. Adipokines are synthesized and secreted by either adipocytes or preadipocytes, endothelial and immune cells, fibroblasts, or other cell types within adipose tissue [12,13]. Adipokines act on nearby or remote tissues via paracrine, autocrine, and endocrine mechanisms, and alternations in adipokine secretion may contribute to obesity-related diseases (e.g., cancers, hypertension) [12,13]. Accumulating evidence indicates that adipokines are important players in the pathogenesis of PH [[14], [15], [16]]. Alternations in the production and secretion of adipokines (e.g., leptin and adiponectin) have been observed in PH patients and rodents PH models. Several adipokines, including leptin, visfatin and resistin, have been demonstrated to display pro-inflammatory and pro-proliferation properties and contribute to the progression of PH [14,17,18]. However, other adipokines, including adiponectin, omentin and fibroblast growth factor 21 (FGF21), exert beneficial effects by attenuating pulmonary vascular remodeling of PH [[19], [20], [21]]. Thus, imbalanced production of adipokines caused by obesity may contribute to the development of PH.

Although, several excellent reviews have discussed the roles of several adipokines in the progression of PH, but only a small fraction of adipokines, including adiponectin, leptin, resistin, omentin, apelin, are mentioned [10,11,[22], [23], [24], [25]], and there is a lack of exploration of recently discovered adipokines. Hence, in this review, we aim to provide a comprehensive summary of the critical influence of selected adipokines on the development of PH. These findings may contribute to a better understanding of these adipokines that allows the establishment of appropriate therapeutic strategies to counteract obesity-associated PH.

## 2 Detrimental roles of adipokines in PH

### 2.1 Leptin

Leptin (Ob), a 16 kDa nonglycosylated peptide encoded by the obese gene (*ob*), is the most extensively studied adipokine thus far, and is predominantly secreted from adipose tissue. Obese and overweight patients often have hyperleptinmia [26,27]. Leptin plays a key role in regulating a variety of physiological functions such as energy metabolism, glycemic control, angiogenesis, neuroendocrine function, immunity, and the inflammatory response [[26], [27], [28]]. The action of leptin mainly relies on leptin receptor (ObR) autophosphorylation, which triggers multiple signal transduction pathways for Janus kinase-signal transducer and activator of transcription-3 (JAK-STAT3), insulin receptor substrate (IRS)/phosphatidylinositol 3 kinase (PI3K), mitogen-activated protein kinase (MAPK), extracellular signal-regulated protein kinase (ERK), and 5′-adenosine monophosphate-activated protein kinase (AMPK) [27]. Increasing evidence suggests that dysfunction of the Ob/ObR axis is involved in various pathological conditions and diseases (e.g., Type 2 diabetes mellitus (T2DM), hypertension, atherosclerosis, and cancers) [[29], [30], [31], [32]]. For example, overactivation of the Ob/ObR axis can enhance the proliferation and migration of vascular smooth muscle cells, modulate inflammatory cell infiltration, and promote cytokine secretion [32,33].

Similarly, Ob/ObR axis has also been demonstrated to be involved in the vascular remodeling of PH [14,34]. Elevated circulating levels of leptin were observed in idiopathic pulmonary arterial hypertension (IPAH) and connective tissue disease-associated pulmonary arterial hypertension (CTD-PAH) patients and murine models of PH [14,34,35], and the expressions of both Ob and ObR were also found to be upregulated in the pulmonary vasculature of IPAH patients and experimental PH mice [14,36]. Additionally, the mean pulmonary arterial pressure (mPAP), right ventricle hypertrophy (RVH), and pulmonary vascular remodeling of hypoxia-induced PH are attenuated in leptin-deficient (*ob/ob*) mice [14,35,37]. Overproliferation and resistance to apoptosis of PASMCs are the predominant factors driving pulmonary vascular remodeling [35]. In IPAH patients, PASMCs express more ObR than that of controls and display more proliferative activities after exogenous administration of leptin [14]. Similarly, in vitro, leptin can stimulate PASMCs proliferation in a concentration and time dependent manner [35], and downregulation of peroxisome proliferator-activator receptor γ (PPARγ) and activation of ERK/STAT-3/Akt1 phosphorylation pathway may contribute to leptin-induced enhancement of PASMCs proliferation [35,38]. Furthermore, Ob/ObR axis has also been shown to play a pivotal role in the immune dysregulation of PH. Activation of the macrophages/monocytes lineage plays a critical role in the development of PH [39]. In IPAH patients, activated macrophages/monocytes also overexpress ObR than that of controls, and perivascular macrophages/monocytes lineage are markedly activated by treatment with leptin [14]. Moreover, ObR expression on the regulatory T-cell (Treg) membrane was markedly upregulated in IPAH and CTD-PAH, and leptin can inhibit the function of Treg of IPAH and CTD-PAH patients and hypoxia-induced PH rats, and ObR deficient rats were protected against Treg dysfunction in hypoxia-induced PH [34,40]. Taken together, these findings suggest that the Ob/ObR axis exerts detrimental effects on the progression of PH by acting as a pro-proliferative and immunomodulatory mediator.

However, several contradictory evidence suggest that the deleterious role of Ob/ObR axis is questionable. Under normoxic conditions, *ob/ob* mice spontaneously pulmonary hypertension and exhibit many characteristics of pulmonary vascular pathology of PH, including increased mean right ventricular systolic pressure (RVSP) and pulmonary arterial wall thickness, proliferation of inflammatory and fibrotic cell types, and altered extracellular matrix deposition [6,41]. Similarly, mild increment of mPAP, RVH and pulmonary artery thickening has been observed in Zucker diabetic fatty rats which are lacking ObR [42,43]. However, data concerning the relationship between pulmonary hypertension and leptin receptor-deficient (*db/db*) mice is lacking, as employed in systemic vasculature, various methods (e.g., noninvasive ultrasound and pulse wave velocity) can be used to assess the pulmonary hemodynamics in *db/db* mice with PH in further studies [44,45]. Additionally, in PAH patients, when corrected by body mass index (BMI), low circulating levels of leptin are associated with an increased overall mortality and the leptin/BMI ratio represented a negative predictive value for mortality at 2 years [46]. Thus, more studies are needed to further investigate the roles and underlying mechanisms of the Ob/ObR axis in PH.

### 2.2 Resistin

Resistin (also known as adipocyte secreted factor (ADSF), C/EBP-epsilon-regulated myeloid-specific secreted cysteine-rich protein (XCP1), or found in the inflammatory zone (FIZZ3)) is a small cysteine-rich secreted and circulating polypeptide that was first discovered in rodents as a novel adipokine with insulin resistance properties [22,47]. Circulating levels of resistin are elevated in rodent obesity models [47]. Resistin is a founding member of the resistin-like molecule (RELM) family, which also includes RELMα, RELMβ, and RELMγ. To date, four RELM family genes (*Retn, Retnla, Retnlb,* and *Retnlg*) have been discovered in rodents and most mammals, however, only two RELM family genes (*Retn* and *Retnlb*) have been discovered in humans. Human resistin (hResistin) is highly expressed in adipose tissue from obese subjects (but not lean subjects) [48], bone marrow, immune cells, and lung tissues [49]. However, hResistin shows a greater similarity in expression pattern and functions to murine/rodent RELMα (mRELMα, also called hypoxia-induced mitogenic factor, HIMF) than to murine/rodent Resistin (mResistin) [45]. mRELMα and its human homolog, hResistin, have been shown to play a crucial role in the pathogenesis of PH [22,[50], [51], [52], [53]].

Of all RELM family proteins, mRELMα/hResistin has attracted the most attention as a promising therapeutic target against PH. mRELMα is significantly upregulated in the pulmonary vasculature of chronic hypoxia-induced PH models [51]. Similarly, the expression of hResistin is also significantly increased in the lungs of patients with IPAH [52], and elevated serum hResistin levels in systemic sclerosis (SSc) patients also correlate with elevated RVSP [53]. Knockdown of mRELMα can substantially mitigate the development of PH in rats exposed to chronic hypoxia, and transtracheal delivery of the mRELMα gene by adeno-associated virus (AAV) or knockin of hResistin in normal rats causes vascular remodeling and hemodynamic changes similar to those of PH [51,54], suggesting that mRELMα/hResistin is a key contributor to PH pathogenesis. At the cellular and molecular levels, mRELMα/hResistin has mitogenic, angiogenic, vasoconstrictive, inflammatory, and chemokine-like properties [18,55]. mRELMα directly induces the hyperproliferation of PASMC by activating the PI3K/Akt pathway and enhancing calcium-sensing receptor (CaSR) activity [51,56]. mRELMα can also enhance the proliferation and migration of pulmonary microvascular endothelial cells (PMVECs), promote pulmonary artery constriction and stimulate pro-inflammatory and proangiongenic mediator (e.g., VEGF and monocyte chemotactic protein-1, MCP-1) production in PMVECs as well as increasing the production of reactive oxygen species (ROS) by monocytes/macrophages [18,55]. Furthermore, mRELMα has been shown to enhance vascular remodeling, immune cell recruitment, and pro-inflammatory mediator (e.g., IL-6) production by upregulating the expression of HIF-1α in lungs [57]. Additionally, recent studies found that mRELMα/hResisitin can also stimulate the production and release of high mobility group box 1 (HMGB1) in PMVECs and macrophages, and HMGB1 serves as a key damage-associated molecular pattern (DAMP) mediator that subsequently induces PASMC overproliferation, promotes cell autophagy, and inhibits bone morphogenetic protein receptor 2 (BMPR2) expression and activation of immune cells, all of which synergistically contribute to PH pathogenesis [51,52]. However, since mRELMα/hResisitin can also be synthesized and secreted by other tissues and cell types (e.g., lung tissues and immune cells) [22,55], thus, more studies are needed to further determine the roles of adipose tissue-derived mRELMα/hResisitin in the pathogenesis of PH.

### 2.3 NAMPT

Nicotinamide phosphoribosyltansferase (NAMPT, EC 2.4.2.12), also termed visfatin and pre-B-cell colony-enhancing factor (PBEF), was initially discovered as a novel adipokine expressed predominantly in visceral adipose tissues [58], and it was later found to have ubiquitous expression in subcutaneous and perivascular adipose tissues, immune cells, liver, and skeletal muscle [[59], [60], [61]]. In mammals, two distinct forms of NAMPT are currently acknowledged: intra- and extracellular NAMPT (iNAMPT and eNAMPT, respectively). iNAMPT is the rate-limiting enzyme for mammalian nicotinamide adenine dinucleotide (NAD) biosynthesis, and eNAMPT is considered a pro-inflammatory cytokine-like molecule. Under non-inflammatory conditions, NAMPT predominantly exhibits a granular pattern within the nucleus [62]. It is secreted by endothelial cells upon IL-1β stimulation [62,63] and abundantly secreted in maternal circulation [64] and inflammatory conditions such as inflammatory bowel disease (IBD) [65,66]. Elevated circulating levels of NAMPT were detected in patients who were overweight/obese and had T2DM, metabolic syndrome, and cardiovascular diseases [[67], [68], [69]]. In addition, there is numerous evidence for the implication of NAMPT in the pathogenesis of PH [17,70,71].

The expression of NAMPT is markedly upregulated in plasma, lungs and PMVECs isolated from patients with IPAH, and the plasma eNAMPT concentration shows a positive correlation with PH severity [70,71]. Similarly, upregulation of NAMPT is also observed in the plasma and lungs of rodent chronic hypoxia-, MCT- and Sugen/hypoxia-induced PH models [70,71]. Knockout of NAMPT or inhibition of NAMPT by drugs (e.g., FK866 or eNAMPT-neutralizing monoclonal/polyclonal antibody) can significantly attenuate the development of several rodent models of PH [17,70,71]. At the cellular and molecular levels, several physical (e.g., hypoxia) and chemical (e.g., VEGF) PH-relevant stimuli can significantly enhance NAMPT promoter activity in PMVECs and promote NAMPT synthesis and secretion [70,71]. Elevated levels of eNAMPT can promote PASMC proliferation and migration, and confer resistance to apoptosis in a paracrine fashion by enhancing store operated Ca^2+^ entry (SOCE) [70]. Furthermore, elevated levels of eNAMPT can also promote the proliferation of PMVECs and increase endothelial-to mesenchymal transition (EndMT), a pathological process that plays a pivotal role in pulmonary vascular remodeling in both patients and animals with PH [71]. Additionally, microRNA-410 (miR-410) can also mitigate pulmonary vascular remodeling of hypoxia-induced PH by downregulation of NAMPT [72]. Taken together, these findings indicate that NAMPT, particularly eNAMPT, may be a promising therapeutic target for the treatment of PH.

### 2.4 Chemerin

Chemerin, as known as retinoic acid receptor responder 2 (RARRES2), tazarotene induced gene 2 (TIG2), and RAR-responsive protein TIG2, is a recently discovered multifaceted adipokine that is more highly expressed in perivascular adipose tissues than in visceral or subcutaneous adipose tissue and has been shown to regulate adipogenesis, inflammation, angiogenesis and energy metabolism [73]. Elevated gene expression of chemerin and its main receptor ChemR23 (also referred as chemokine-like receptor 1, CMKLR1) are positively correlated with the severity of several diseases such as obesity, T2DM, rheumatoid arthritis and cardiovascular disease [73,74]. Growing evidence suggests that the chemerin/CMKLR1 axis may be implicated in the development of PH [[75], [76], [77]].

Plasma chemerin concentrations are significantly elevated in patients with IPAH, and the expressions of chemerin and CMKLR1 are significantly upregulated in the plasma and lung tissues of rats with MCT-induced PH [[75], [76], [77]]. In vitro treatment with chemerin alone and/or in the presence of endothelin-1 promotes the proliferation and migration of PASMCs by activating the ERK1/2 pathway and confers resistance to apoptosis in rat PASMCs [75,76]. And PH-relevant stimuli (e.g., hypoxia and endothelin-1) can significantly upregulate the expression of CMKLR1 in rat PASMCs [76], which can contribute to the sustained pro-proliferative effects of chemerin. Furthermore, a recent study also reported that chemerin-9 (the active fragment of chemerin)-induced contraction was significantly enhanced in intrapulmonary artery from MCT-treated rats compared with control rats, which may exacerbate the progression of PH [77]. However, more clinical studies are needed to further illustrate the role and underlying mechanisms of the chemerin/CMKLR1 axis in the progression of PH.

### 2.5 Lipocalin 2

Lipocalin 2 (LCN2), also called neutrophil gelatinase-associated lipocalin (NGAL) and oncogene 24p3, is a 25 kDa glycoprotein recently identified as a novel adipokine [78]. This adipokine has been reported to exert pleiotropic effects on iron transport, tumorigenesis, inflammatory response, cell proliferation and oxidative stress and is involved in various pathological conditions and diseases (e.g., obesity, diabetes, cancers and cardiovascular diseases) [79,80]. Circulating LCN2 levels are higher in obese subjects and rodent obesity models [78,81]. Recent studies have indicated that LCN2 plays an adverse role in the pathogenesis of PH [[82], [83], [84]]. Expressions of LCN2 in the plasma and/or lung tissues are markedly upregulated in the MCT-induced and Kawasaki disease-associated PH rats [[82], [83], [84]], as well as increased in patients with congenital heart disease-associated PH (CHD-PH) [82]. In human PASMCs (HPASMCs), upregulation of LCN2 promotes endoplasmic reticulum stress and proliferation by augmenting intracellular iron, activating the PI3K/Akt pathway, and conferring resistance to oxidative stress-induced apoptosis by decreasing intracellular ROS production and increasing the expression of superoxide dismutases 1 and 2 (SOD1 and SOD2) [82,85]. These findings indicate that targeting LCN2 may be a promising therapeutic strategy for the treatment of PH.

### 2.6 Gremlin-1

Gremlin-1 (as known as cysteine knot superfamily, homolog), a secreted glycoprotein, is an important BMPs antagonist and was recently identified as a novel adipokine that plays key roles in regulating cell differentiation and survival, inflammation and organ development [86]. Gremlin-1 is highly expressed in (pre)adipocytes, and the secretion of gremlin-1 is markedly increased in patients with hypertrophic obesity [[86], [87], [88]]. The biological action of gremlin-1 is exerted by binding BMPs (e.g., BMP2 and BMP4), thus inhibiting BMP signaling. BMPs, together with their antagonists, are responsible for maintaining the homeostasis and functions of normal pulmonary circulation. Suppression of BMP signaling is a critical pathological mechanism in the development of PH, and emerging evidence suggests that enhancement of BMP signaling by inhibition of gremlin-1 is a potential therapeutic approach for combating PH [15,88,89].

The expression of gremlin-1 is markedly upregulated in the plasma and lungs of patients with CHD-PH, and the plasma concentration of gremlin-1 shows a positive correlation with CHD-PH severity [90]. Furthermore, elevated gremlin-1 expression is also found in pulmonary endothelium of patients with IPAH and hereditary PAH [15,88,91]. Similarly, elevated expression of gremlin-1 was also found in the plasma, lung tissues and pulmonary arterial endothelial cells (PAECs) isolated from several experimental rodent PH models [90]. Additionally, in mice, haplodeficiency of gremlin-1 mitigates the pulmonary arterial remodeling of hypoxia-induced PH [15]. Treatment with gremlin-1 neutralizing antibodies also mitigated the progression of Sugen/hypoxia-induced PH in mice [88]. Furthermore, in vitro, exposure of PAECs to hypoxia can increase NADPH oxidase 1 (Nox1)-derived ROS production, which promotes nuclear translocation and phosphorylation of CREB, and in association with redox Factor 1 (Ref-1), leads to upregulation of gremlin-1 expression [92]. Upregulated gremlin-1 further enhances the proliferation, migration, and angiogenesis of PAECs and induces PAEC EndMT as well as potentiates the proliferation and inhibits the apoptosis of PASMCs, thereby exacerbating the progression of PH [89,90,[92], [93], [94]]. However, it is still unknown whether inhibition of gremlin-1 can also exert beneficial effects on patients with PH.

### 2.7 DPP-4

Dipeptidyl peptidase-4 (DPP-4, EC 3.4.14.5), also known as CD26, is a novel adipokine that correlates with adipocyte inflammation and insulin resistance [[95], [96], [97]]. Upregulation of DPP-4 has been observed in the visceral adipose tissue of obese patients compared with lean subjects [97]. DPP-4 plays a primary role in cleaving incretin hormones such as glucose-dependent insulinotropic polypeptide (GIP) and glucagon-like peptide 1 (GLP-1). By cleaving incretins, DPP-4 decreases the secretion of insulin and increases glucagon secretion, thereby elevating blood glucose levels. DPP-4 inhibitors (e.g., sitagliptin and linagliptin) have been developed as a novel class of antidiabetic drugs and have therapeutic potential in obesity, metabolic syndrome, and cardiovascular disease [98,99]. Recently, emerging evidence suggests that DPP-4 may be an important therapeutic target for the treatment of PH. DPP-4 expression is upregulated in the lung tissues of rats with hypoxia- and MCT-induced PH [[100], [101], [102]]. In vivo, treatment with a DPP-4 inhibitor (sitagliptin) can significantly attenuate RVSP, right ventricle and pulmonary vascular remodeling, inflammatory cell infiltration, and EndMT in MCT-, bleomycin- and hypoxia-induced PH rats, and treatment with a GLP-1 antagonist can abolish the beneficial effects of sitagliptin on PH [[100], [101], [102]], which suggests that similar to its action on diabetes, DPP-4 inhibitors can also exert protective effects by inhibiting GLP-1 cleavage. Additionally, other mechanisms also contribute to the protective effects of DPP-4 inhibitors on PH. In vitro, sitagliptin alleviated platelet-derived growth factor isoform BB (PDGF-BB)-induced overproliferation of HPASMCs by inhibiting Akt/MAPK pathways and reduced PDGF-BB-induced migration of HPASMCs [101]. However, more investigations are needed to explore the role and underlying mechanism of DPP-4 in PH patients.

### 2.8 SPARC

Secreted protein acidic and rich in cysteine (SPARC), also known as osteonectin and basement-membrane protein 40 (BM-40), is a 34 kDa matricellular glycoprotein and a newly identified adipokine that is mainly secreted by adipocytes [103]. The expression of SPARC is dramatically upregulated in the adipose tissue of patients with obesity and in obese rodent models [104,105], and circulating levels of SPARC are also elevated in patients with obesity and T2DM and show a positive correlation with BMI and insulin resistance [106,107]. As a multifunctional extracellular matrix (ECM)-binding protein, adipose tissue-derived SPARC plays key roles in regulating angiogenesis, the inflammatory response, tissue remodeling and fibrosis [103,108]. Recently, evidence suggested that SPARC may be a potential therapeutic target for the treatment of PH [16]. SPARC expression is significantly upregulated in the lung tissues of patients with IPAH and mice with hypoxia-induced PH. Similarly, in vitro, hypoxia exposure significantly increased the expression of SPARC in human PASMCs by activating the HIF-2α and TGF-β1 signaling pathways [16]. Moreover, in vitro, the proliferation of PASMCs is potentiated by recombinant SPARC and inhibited by knockdown of SPARC, and AAV-mediated knockdown of SPARC can significantly improve hemodynamic and cardiac function in PH mice [16]. However, the exact role underlying mechanism of SPARC in PH remains unclear.

## 3 Beneficial roles of adipokines in PH

### 3.1 Adiponectin

Adiponectin (APN), as called GBP-28, apM1, AdipoQ and Acrp30, a 30 kDa secretory protein, one of the most extensively studied adipokine that is produced and mainly secreted by adipose tissue and shows pleiotropic effects with insulin-sensitizing, anti-inflammatory, and anti-fibrotic properties by interacting with adiponectin receptor 1 and 2 (AdipoR1/R2) [109,110]. Circulating levels of APN are negatively correlated with BMI and often downregulated in obese patients, T2DM, and cardiovascular diseases [[109], [110], [111]]. Recent studies reported that reduced expressions of APN in the plasma and pulmonary vasculature were observed in obesity-induced PH mice and circulating levels of APN show a negative correlation with the elevated RVSP [112,113], but more evidence shown that circulating levels of APN and/or lung APN expressions are higher in patients with PH (e.g., IPAH and CHD-PH) and rodents experimental PH models than their controls [[114], [115], [116], [117]].

Moreover, APN was demonstrated to exert beneficial effects on multiple pathogenic processes (e.g., vascular remodeling, inflammatory response, and insulin resistance) underlying PH development [10,19,113,117,118]. In vivo, under hypoxia or ovalbumin exposure, elevated pulmonary arterial pressure, more severe pulmonary arterial muscularization, and greater pulmonary arterial remodeling are observed in APN knockout (APN^−/−^) mice [19,117,118]. Overexpression of APN or exogenous administration of APN can mitigate the development of experimental PH models [19,118]. In vitro studies also showed that APN can inhibit the proliferation of PASMCs by suppressing the serum response factor-serum response element (SRF-SRE) pathway [19]. Moreover, several growth factors such as PDGF-BB, basic FGF, and VEGF are increased in pulmonary artery of PH patients and murine PH models, leading to PASMC overproliferation and pulmonary arterial muscularization and vascular remodeling. APN was also found to bind with these growth factors, which may also mitigate overproliferation by sequestering its receptor in PAMSCs [119,120]. In addition, APN has endothelium-dependent vasodilator properties. Endothelial NOS (eNOS, a potent vasodilator) and NO production were reduced in APN^−/−^ mice, which may affect the pulmonary vascular tone of PH patients [121]. Noteworthy, APN also has potent anti-inflammatory effects on perivascular immune cell infiltration by inhibiting the mTOR and NF-κB pathways and suppressing immune cell-endothelial cell interactions by downregulating E-selectin in PAECs [119]. APN has also been reported to inhibit eosinophil accumulation around the pulmonary vasculature by downregulation of macrophage-derived chemokines, which may attenuate the progression of eosinophoil inflammation (e.g., schistosomiasis)-induced PH [122]. Furthermore, systemic insulin resistance (i.e., increased fasting blood glucose and insulin levels) is increasingly implicated in the pathophysiology of PH [108,123,124]. APN serves as an insulin-sensitizing adipokines that has direct effects on glycemic control (decreased glucose production and increased peripheral glucose uptake) and lipid handling (decreased lipolysis, downregulation of lipogenesis, and increased fatty acid beta (β)-oxidation) [125,126], whether APN contributes to the insulin resistance of PH requires further investigation. In conclusion, these findings indicate that OPN plays a beneficial role in the progression of PH.

### 3.2 CTRP9

C1q/TNF-related proteins (CTRPs) are a highly conserved family of APN paralogs that share common structural characteristics with C1q complement components and TNF receptor ligands. Among the 15 known members of CTRPs (CTRP1∼15), CTRP9 shares the greatest structural similarity with APN and is also highly expressed in adipose tissues [127]. Circulating levels of CTRP9 are decreased in rodent models of obesity [127,128]. After binding with AdipoR1 and N-cadherin, CTRP9 activates various signaling pathways (e.g, AMPK, ERK signaling pathways) to regulate glucose and lipid metabolism, vasodilation and cell differentiation [128,129]. Similar to APN, CTRP9 also serves as an insulin-sensitizing and anti-inflammatory adipokine that exerts protective effects against several diseases, including diabetes, cardiovascular disease and stroke [128]. Recently, emerging evidence suggests that CTRP9 may also exert a beneficial effect on the development of PH [130,131].

The expression of CTRP9 is markedly decreased in plasma and PMVECs isolated from patients with PH, and the plasma CTRP9 concentration shows a negative association with PH severity [132]. Similarly, decreased expression of CTRP9 in plasma, lung tissue and PASMCs was observed in hypoxia- and arteriovenous shunt-induced PH models [[130], [131], [132]]. Transtracheal delivery of the CTRP-9 gene by AAV or siRNA can decrease RVSP and attenuate the right ventricle and pulmonary vascular remodeling and inflammation of hypoxia- and arteriovenous shunt-induced PH [130,131]. Additionally, in vitro experiments also demonstrated that CTRP9 exerts anti-proliferative effects on hypoxia-treated PASMCs by inhibiting of TGF-β1/ERK1/2 signaling, inducing apoptosis and inhibiting the migration of PASMCs [133]. CTRP9 is also reported to inhibit PMVEC apoptosis via activation of the PI3K/Akt pathway [132]. Furthermore, overexpression of CTRP9 can induce the production of the vasodilator nitric oxide (NO) and decrease vasoconstrictor ET-1 production by activating eNOS and inhibiting AMPK/ERK1/2 pathway in PMVECs, which may contribute to CTRP9-induced endothelium-dependent vasodilation in hypoxia-induced PH [131].

### 3.3 FGF21

Fibroblast growth Factor 21 (FGF21) is a ∼22 kDa protein belonging the FGF superfamily, which is mainly produced by adipose tissue, the liver, and the pancreas [134]. Unlike conventional FGF protein, FGF21 acts as an endocrine hormone with pleiotropic effects on glucose homeostasis, lipid metabolism and energy balance and without mitogenic activities. Circulating levels of FGF21 are markedly increased in patients with obesity, T2DM and metabolic syndrome [135]. In the cardiovascular system, secretion of FGF21 can protect the heart from hypertrophy, ischemia-reperfusion injury, and oxidative stress [136,137]. In addition to having cardioprotective functions, FGF21 has also been shown to exert protective effects against the development of PH [21,138].

Compared with healthy controls, plasma FGF21 concentrations are markedly increased in IPAH and HPH patients [115,139] as well as increased in rodent MCT-, hypoxia- and Sugen/hypoxia-induced PH models [115], and the vascular remodeling of hypoxia-induced PH can be attenuated by exogenous administration of FGF21 and exacerbated in FGF21 knockout mice [21,140], indicating that FGF21 may be a protective factor against PH. Several mechanisms have been demonstrated to explain the beneficial effects of FGF21 on the progression of PH. In vitro, FGF21 can significantly upregulate the expression of peroxisome proliferator-activator receptor γ (PPARγ) by downregulating microRNA-130 and microRNA-27b [141,142] and activating the AMPK/PPARγ coactivator-1α (PGC-1α) pathway [140]. PPARγ is an important transcription factor that has been shown to inhibit PASMC proliferation, suppress the inflammatory response and attenuate endothelial dysfunction. Evidence suggests that upregulated PPARγ may contribute to the protective effects of FGF21 on hypoxia-induced overproliferation and apoptosis resistance of PASMCs [141]. A recent study also indicated that FGF21 can inhibit PASMC overproliferation by suppressing the mechanistic target of rapamycin complex 1 (mTORC1)/eukaryotic translation initiation Factor 4E–binding protein 1 (EIF4EBP1) pathway by upregulating long noncoding RNA (lncRNA) H19 [21]. Moreover, FGF21 can attenuate hypoxia-induced dysfunction, apoptosis, and inflammation in HPAECs by alleviating endoplasmic reticulum stress and inhibiting the miR-27b/PPARγ pathway [138,139,142]. Additionally, FGF21 can significantly reduce the production of pro-inflammatory cytokines (e.g., TNF-α, IL-1 and IL-6), which may also contribute to the protective effects of FGF21 on the development of PH [143].

### 3.4 Apelin

Apelin (APLN), as an endogenous ligand of the G-protein-coupled receptor APJ (*APLNR*), is a novel adipokine that plays a key role in regulating glucose metabolism, lipolysis, cell proliferation and cardiovascular homeostasis [144]. Apelin is abundantly secreted by adipocytes, and plasma apelin concentrations are elevated in patients with obesity and T2DM [144]. In addition to adipose tissue, the apelin-APJ system is also widely expressed in other tissues, including the lungs, liver, and cardiovascular system [[145], [146], [147]]. The *APLN* gene encodes a 77 amino acid prepropeptide that is released into the circulation in several active fragments, including apelin-12, -13, −16, −17, −19 and −36. Shorter forms of apelin (apelin-13) show much higher biological potency than the longer forms (apelin-36). Extensive evidence has shown that apelin-APJ signaling exerts anti-inflammatory and antioxidant effects on multiple cardiovascular diseases [145]. Recently, evidence has indicated that apelin-APJ signaling is emerging as a novel therapeutic target for PH [146,148].

Both apelin and APJ are highly expressed in the endothelial cells of the pulmonary vasculature. Reduced expression of apelin has been observed in the plasma and pulmonary endothelium of patients with PH as well as the in the lungs of MCT-, hypoxia- and Sugen/hypoxia-induced PH rodent models [146,[148], [149], [150], [151]]. Additionally, under hypoxia exposure, apelin knockout (APLN^−/−^) mice developed more severe PH than wild-type mice and displayed marked pulmonary vascular defects [148]. Treatment with apelin or G protein-biase apelin analogue (e.g., MM07) can decease RVSP and attenuate right ventricle hypertrophy and pulmonary arterial muscularization in PH rodent models [148,152]. In a randomized, double-blind, placebo-controlled clinical trial (NCT01457170), short-term administration of apelin reduced pulmonary artery resistance and increased cardiac output and stroke volume in patients with PH [153]. Collectively, these findings suggest that augmentation of apelin-APJ signaling may be a potential therapeutic approach for PH.

Several signaling pathways and targets have been proposed as mechanisms underlying the beneficial effects of apelin-APJ signaling in PH: downregulation of apelin-APJ signaling in PAECs can lead to a significant reduction in hypoxia-induced AMPK phosphorylation, which subsequently reduces the expression of Kruppel-like factor 2 (KLF2), which in turn can enhance eNOS activities and ultimately result in decreased NO production and exacerbation of PH, thus, augmentation of the apelin-APJ-AMPK-KLF2-eNOS axis may exert therapeutic effects on PH [148]. Furthermore, several miRNAs, including miR-424 and miR-503, have been identified as downstream targets of apelin-APJ signaling in the pathogenesis of PH. Downregulation of apelin-APJ signaling in PAECs leads to a significant increase in the expression of FGF2 and FGF receptor 1 (FGFR1) by inhibiting miR-424 and miR-503 expression. Apelin-induced inhibition of FGF signaling can attenuates the overproliferation of PAECs and PASMCs, which also alleviates the progression of PH [146]. In addition, apelin has been reported to inhibit the overproliferation and migration of PASMCs mediated by activating PI3K/Akt/mTOR signaling as well as promote the proliferation and decrease the apoptosis of PAECs [150,154]. Treatment with small molecule apelin agonist (e.g., CMF-019) or G protein-biase apelin analogue (e.g., MM07) can attenuate the apoptosis of PAECs [155]. Moreover, apelin expression can be enhanced by PPARγ. In rodent PH models, downregulation of PPARγ by suppression of BMPR2 signaling and miR-130/301 can also decreases the apelin expression, which subsequently increases the apoptosis of PAECs and induces overproliferation of PASMCs [150].

### 3.5 Omentin-1

Omentin, as known as intelectin-1 and intestinal lactoferrin receptor, is a ∼34 kDa secretory glycoprotein that was recently identified as a novel insulin-sensitizing adipokine preferentially expressed in visceral adipose tissue [156]. Omentin-1 is the major circulating form of omentin in human plasma, and circulating levels of omentin-1 are decreased in obese/overweight subjects compared with lean subjects [157]. Recently, evidence has also shown that omentin-1 may be a protective factor against PH. Elevated circulating levels of omentin-1 are associated with increased RVSP in patients with SSc [158]. Administration of exogenous omentin-1 can significantly attenuate the hemodynamic changes and RV and pulmonary arterial remodeling of MCT-induced PH [20]. Additionally, Omentin is well demonstrated to exert anti-proliferative, anti-inflammatory, and anti-oxidative effects on cultured vascular endothelial and smooth muscle cells as well as induce vasodilation in rat isolated blood vessel by increasing NO production [[159], [160], [161]], which play a key role in vascular diseases, however, whether these aforementioned mechanisms also contribute to the association between omentin-1 and PH pathogenesis requires further investigations.

### 3.6 FSTL 1

Follistatin-like 1, a 37 kDa secreted glycoprotein, belongs to the SPARC family and is considered to be a novel adipokine that is synthesized and secreted by adipose tissue [162]. Circulating levels of FSTL1 are increased in patients who are overweight, obese or exhibit T2DM compared with healthy lean controls [163,164]. Recently, evidence has suggested that serum FSTL1 levels are markedly increased in patients with PH related to chronic obstructive pulmonary diseases (COPD) and that the lung expression of FSTL1 is upregulated in a hypoxia-induced PH mouse model [165]. In vivo, the malignant phenotype of hypoxia-induced PH is exacerbated in FSTL1 knockout (FSTL1^−/−^) mice and can be attenuated by treatment with recombinant FSTL1 [165]. Moreover, in vitro experiments also demonstrated that FSTL1 exerts direct inhibitory effects on the proliferation and migration of HPASMCs by inhibiting ERK signaling [164]. However, more studies are needed to further confirm the beneficial effect of FSTL1 in PH.

### 3.7 Vaspin

Visceral adipose tissue-derived serpin (vaspin), as known as sepin A12, OL-64, viscer adipose-specific serpin, is a newly identified insulin-sensitizing adipokine in obesity. Vaspin is mainly secreted by visceral adipose tissues, and circulating levels of vaspin are higher in patients with obesity [166]. Accumulating data have shown that vaspin plays protective roles in the pathogenesis of multiple diseases (e.g., diabetes, rheumatoid arthritis, and atherosclerosis) [167]. Recently, evidence has shown that vaspin also exerts beneficial effects in PH [168]. A recent study showed that circulating vaspin levels are decreased in patients with PH. Administration of exogenous vaspin can significantly attenuate the hemodynamic changes and pulmonary arterial fibrosis of MCT-induced PH by inhibiting the ROS/MMP-2/fibrosis pathway [169]. However, the date for vaspin in PH is still scarce and further studies are needed to clarify the beneficial roles and underlying mechanisms of vaspin in PH.

## 4 Undetermined roles of adipokines in PH

### 4.1 PAI-1

Plasminogen activator inhibitor-1 (PAI-1), as known as endothelial plasminogen activator inhibitor and serpin E1, is a novel adipokine that is mainly secreted by adipose tissue, particularly visceral adipose tissue [170], and this adipokine suppresses fibrinolysis by inhibiting tissue- and urokinase-type plasminogen activator (t-PA and u-PA, respectively). Circulating levels of PAI-1 are increased in patients with obesity and metabolic dysfunction syndrome [171], and are independent risk factors for stroke, cardiovascular disease, and obesity-related cancers [[171], [172], [173]]. In addition to its anti-fibrinolytic effects, PAI-1 has been implicated in the progression of PH [[174], [175], [176], [177]].

Evidence is contradictory concerning the role of PAI-1 in the development of PH. Recently, one study reported that the serum concentration of PAI-1 shows a significant positive correlation with the severity of mPAP, and serum levels of PAI-1 may be a useful predictor of severity in patients with PH [177]. Moreover, some studies have shown that PAI-1 expression is upregulated in the lung tissues of the MCT-induced rat PH model [[178], [179], [180], [181]], which can be attenuated by inhibition of TGF-β signaling, suggesting that PAI-1 may serve as a major effector and downstream target of TGF-β signaling in PH. However, other studies have shown that the expression of PAI-1 is downregulated in the lung tissues and PASMCs of patients with IPAH and in a Sugen/hypoxia-induced rat PH model [175,176]. PAI-1 expression can be negatively regulated by miR-17∼92 clusters which are key regulators of PH [182], and overexpression of PAI-1 can attenuate miR-17∼92-induced PASMC hyperproliferation [175], suggesting that PAI-1 is a negative regulator of miR-17∼92 signaling in PH. Collectively, further investigations are needed to confirm the exact roles of PAI-1 in PH.

## 5 Comments and perspective

The dysregulation of adipokines is an important pathological hallmark of obesity and plays critical roles in the development of obesity-related diseases (e.g., atherosclerosis) [12,13]. PH is a vascular remodeling disease linked to systemic and perivascular inflammation, oxidative stress, and insulin resistance [6,113,123]. Accumulating evidence indicates that the dysregulation of adipokines may plays a key role in the pathogenesis of PH [14,71]. In this review, we summarized that several adipokines (e.g., leptin) exert detrimental effects on the progression of PH, whereas other important adipokines (e.g., adiponectin) serve as protective factors against PH. Imbalance of these adipokines leads to the development of low-grade chronic inflammation, insulin resistance and oxidative stress that may contributes to the vascular remodeling of PH. Targeting adipokine dysregulation has been shown to exhibit potential therapeutic effects on PH [[15], [16], [17]].

**Fig. 1:**
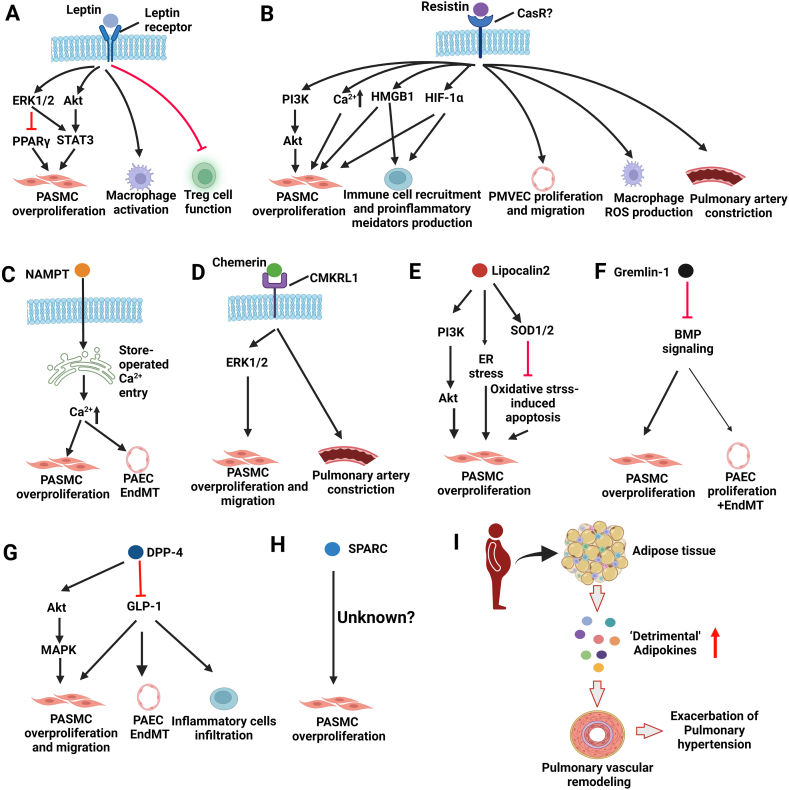
Detrimental roles of selected adipokines in pulmonary hypertension **A-H.** Detrimental roles and underlying mechanisms of Leptin (**A**), Resistin (**B**), NAMPT (**C**), Chemerin (**D**), Lipocalin 2 (**E**), Gremlin-1 (**F**), DPP-4 (**G**) and SPARC (**H**) in the pathogenesis of pulmonary hypertension. **I.** Selected adipokines play detrimental roles in the pulmonary vascular remodeling of pulmonary hypertension. **Abbreviation:** BMP, bone morphogenetic protein; CaSR, calcium-sensing receptor; CMKRL1, chemerin chemokine-like receptor 1; DPP-4, dipeptidyl peptidase-4; ERK1/2, extracellular signal-regulated protein kinase 1/2; EndMT, endothelial to mesenchymal transition. ER stress, endoplasmic reticulum stress; GLP-1, glucagon-like peptide 1, HMGB1, high mobility group box 1; HIF-1, hypoxia inducible factor-1; MAPK, mitogen-activated protein kinase; NAMPT, nicotinamide phosphoribosyltansferase; PASMC, pulmonary artery smooth muscle cell; PAEC, pulmonary arterial endothelial cell; PPAR, peroxisome proliferation-activator receptor; PI3K, phosphatidylinositol 3 kinase; ROS, reactive oxygen species; SOD1/2, superoxide dismutases 1/2; SPARC, secreted protein acidic and rich in cysteine. STAT3, signal transduce and activator of transcription 3; Treg, regulatory T cell (Figures are created using Biorender. com).

**Fig. 2:**
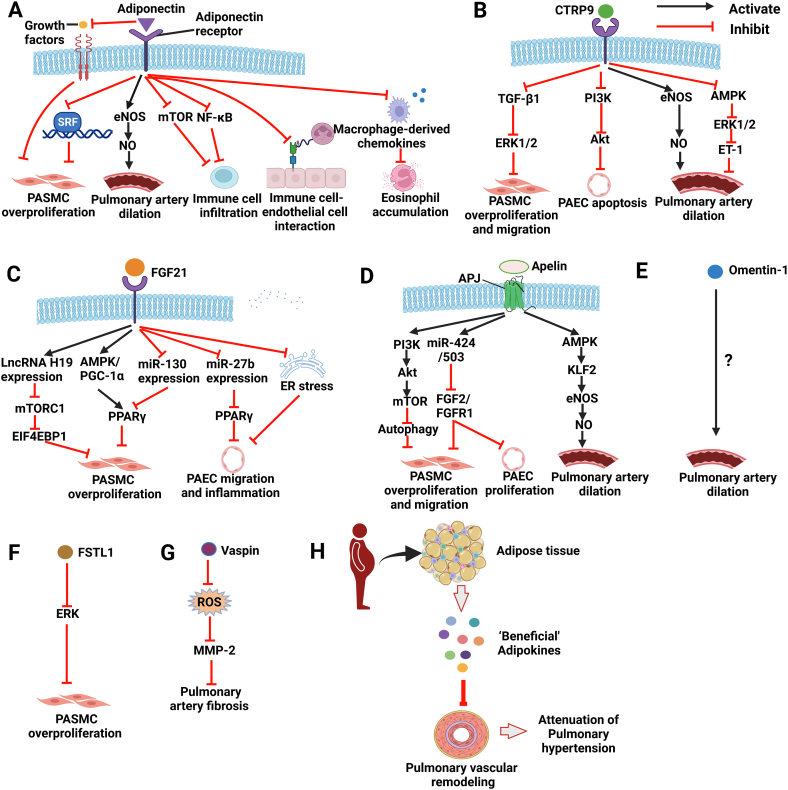
Beneficial roles of selected adipokines in pulmonary hypertension **A-G.** Detrimental roles and underlying mechanisms of Adiponectin (**A**), CTRP9 (**B**), FGF21 (**C**), Apelin (**D**), Omentin-1 (**E**), FSTL1 (**F**) and Vaspin (**G**) in the pathogenesis of pulmonary hypertension. **H.** Selected adipokines play detrimental roles in the pulmonary vascular remodeling of pulmonary hypertension. **Abbreviation:** AMPK, 5′-adenosine monophosphate-activated protein kinase; APJ, apelin receptor; CTRP9, C1q/TNF-related protein 9; eNOS, endothelial nitric oxide synthase; EIF4EBP1, eukaryotic translation initiation Factor 4E–binding protein 1; ER stress, endoplasmic reticulum stress; ERK1/2, extracellular signal-regulated protein kinase 1/2; ET-1, endothelin-1; mTORC, mechanistic target of rapamycin complex 1; FGF21; fibroblast growth factor 21; FGFR1, fibroblast growth factor receptor 1; FSTL1, follistatin-like 1; KLF2, krupple-like factor 2; MMP-2, matrix metalloproteinas-2; PASMC, pulmonary artery smooth muscle cell; PAEC, pulmonary arterial endothelial cell; PPARγ, peroxisome proliferation-activator receptor γ; PGC-1α, PPARγ coactivator--1α; PI3K, phosphatidylinositol 3 kinase; SRF, serum response factor; TGF-1, transforming growth factor 1; ROS, reactive oxygen species (Figures are created using BioRender.com).

Current available evidence suggests that circulating levels of 8 adipokines such as leptin, chemerin and SPARC are elevated in obese humans and rodent models and upregulated in the plasma and/or lung tissues of patients with PH and rodent PH models [16,17]. The upregulation of these adipokines is reported to exert detrimental effects by facilitating the development of PH, including promoting PASMC overproliferation, conferring resistance to apoptosis of PASMCs, increasing oxidative stress, inducing EndMT and regulating immune cell functions (summarized in Fig. 1 and Table 2). Additionally, the hemodynamic changes and pulmonary arterial remodeling of PH are significantly attenuated by inhibition of these adipokines. However, 7 key adipokines (e.g., adiponectin, FGF21, CTRP9 and omentin-1) are suggested as protective factors against PH and are reported to inhibit PASMC overproliferation, induce endothelium-dependent vasodilation, suppress the inflammatory response, thereby mitigating the progression of PH (summarized in Fig. 2 and Table 2). Exogenous administration of these adipokines exerts therapeutic effects on the development of PH [130]. Notably, these ‘beneficial’ adipokines are decreased (e.g., adiponectin, CTRP9, FGF21 and omentin-1) and increased (e.g., apelin, FSTL1) in the plasma and/or lung tissues of PH patients and/or rodent PH models, which is not fully consistent with these changes in obese humans and animals. Adipokines (e.g., FGF21) are not only secreted by adipose tissue but can also be secreted by other tissues, such as the liver, heart, lungs, and brain [183]. Additionally, to date, evidence is insufficient to determine the exact roles of several adipokines (e.g., PAI-1) in PH. Thus, more investigations are needed to further determine the roles of adipose tissue-derived adipokines in PH.

**Table 2:** Roles of selected adipokines in pulmonary hypertension (PH).

Adipokines	Circulating levels in Obese patients	PH Patients	Rodent models of PH	Functional Roles in PH
Leptin	↑	Plasma↑, lung↑ Pulmonary vasculature↑	Plasma↑, lung↑ Pulmonary vasculature↑	Promotes PASMC proliferation, enhances marcophage activation, inhibits Treg cell function and exacerbates PH [14,35,41]
Resistin	↑	Plasma↑, lung↑	–	Promotes the proliferation of PASMC and PMVEC, facilitates PMVEC migration, increases immune cell recruitment and the productions of proinflammatory meidators and ROS, enhances pulmonary artery constriction, and aggravates pulmonary vascular remodeling [51,52,[54], [55], [56], [57]]
NAMPT	↑	Plasma↑, lung↑, PMVECs↑	Plasma↑, lung↑	Promotes PASMC proliferation and migration, enhances PMVEC proliferation and EndMT [70,71]
Chemerin	↑	Plasma↑	Plasma↑, lung↑	Promotes PASMC proliferation and enhances pulmonary artery constriction [[75], [76], [77]]
Lipocalin2	↑	Plasma↑	Plasma↑, lung↑	Promotes PASMC proliferation [82,85]
Gremlin-1	↑	Plasma↑, lung↑	Plasma↑, lung↑	Promotes the proliferation of PASMC and PAEC, enhances PAEC EndMT, and aggravates pulmonary vascular remodeling [89,90,[92], [93], [94]]
DPP-4	↑	–	Lung↑	Promotes PASMC proliferation and migration, enhances PAEC EndMT, facilitates inflammatory cells infiltration and aggravates pulmonary vascular remodeling [[100], [101], [102]]
SPARC	↑	Lung↑	Lung↑	Promotes PASMC proliferation [16]
Adiponectin	↓	Plasma↑, lung↑ [[114], [115], [116], [117]]	Plasma↓, Pulmonary vasculature↓ [112,113] Plasma↑, lung↑ [[114], [115], [116], [117]]	Inhibits PASMC proliferation, suppresses immune cell infiltration and immune cell-endothlial cell interaction, induces pulmonary artery dilation, and mitigates pulmonary vascular remodeling [19,[119], [120], [121], [122]]
CTRP9	↓	Plasma↓	Plasma↓	Inhibits PASMC proliferation and migration, suppresses PMVEC apoptosis, and induces pulmonary artery dilation [[131], [132], [133]]
FGF21	↑	Plasma↑ [115,139]	Plasma↑ [115]	Inhibits PASMC proliferation, suppresses PAEC migration and pro-inflammatory mediators production [21,138,[140], [141], [142], [143]]
Apelin	↑	Plasma↓, Pulmonary vasculature↓	Plasma↓, Pulmonary vasculature↓	Inhibits PASMC proliferation and migration, suppresses PMVEC proliferation, induces pulmonary artery dilation, and mitigates pulmonary vascular remodeling [148,150,154,155]
Omentin-1	↓	Plasma↑	–	Induces pulmonary artery dilation [20]
FSTL1	↑	Plasma↑	Lung↑	Inhibits PASMC proliferation and migration [164,165]
Vaspin	↑	Plasma↓	–	Inhibits pulmonary artery fibrosis [169]
PAI-1	↑	Plasma↑ [171]	Lung↓ [175,176] lung↑ [[178], [179], [180], [181]]	Inhibits PASMC proliferation [175] As a predictor of severity in PH patients [177]

PASMC, pulmonary artery smooth muscle cell; PAEC, pulmonary arterial endothelial cell; PMVEC, pulmonary microvascular endothelial cells; ROS, reactive oxygen species; EndMT, endothelial to mesenchymal transition.

Over recent decades, substantial progress has greatly expanded our knowledge about relationship between adipokines and PH. However, there are several gaps in understanding the roles and underlying mechanisms of adipokines in the pathogenesis of PH: (1) Currently, adipose tissue is known to secrete more than 600 proteins, and only a small proportion of these has been investigated [12]. More investigations are needed to further analysis the secretome of adipose tissue from patients with PH, and whether other novel adipokines (e.g., FABPs: fatty-acid binding proteins; SPARCL1: SPARC-like protein 1) are involved in the pathogenesis of PH [184,185] (2) In addition to adipose tissue, adipokines (e.g., FGF21). can be synthesized and released in other tissues (e.g., liver, lung, and skeletal muscles) [183], other tissues-derived adipokines may also affect the progression of PH. Thus, in further studies, experiments are performed in adipose tissue-specific conditional knockout of adipokines in mice may be more convincing to evaluate the precise roles and underlying mechanisms of adipokines in PH. (3) Evidence regarding the effects of the agonists/antagonists of adipokines on the progression of PH are limited, additional high-quality clinical trials are needed to confirm the therapeutic effects of these drugs in PH patients. (4) Given the complex multifactorial nature of PH, no single animal model can per se completely mimic the pathogenesis of human PH. For example, mouse PH models induced by either chronic hypoxia or Sugen/hypoxia exhibit severe vascular remodeling but fail to induced stable vascular occlusion and complex plexiform-like lesions, none of them fully resemble the pathology of clinical PH. Hence, more rodent models of PH are needed to provide a comprehensive understanding of the roles and underlying mechanisms of adipokines in the pathogenesis of human PH [186,187].

## 6 Conclusions

In conclusion, adipose tissues produce various adipokines that function to regulate the proliferation and apoptosis resistance of pulmonary vascular cells, endothelial function and EndMT, inflammation, and oxidative stress. Obesity-induced dysregulation of adipokines leads to systemic low-grade inflammation, insulin resistance and oxidative stress, which may affect the vascular remodeling process in PH patients. Targeting dysregulated adipokines appears to be a potential novel therapeutic strategy for the treatment of PH.

## Data availability statement

No data was used for the research described in the article.

## Funding

This work was supported by grant 82002100 (to Yiyi Yang) from the 10.13039/501100001809National Natural Science Foundation of China (Beijing, China).

## CRediT authorship contribution statement

**Qi Jia:** Writing – review & editing, Writing – original draft, Conceptualization. **Yeling Ouyang:** Writing – original draft. **Yiyi Yang:** Writing – original draft, Funding acquisition. **Shanglong Yao:** Writing – review & editing, Writing – original draft. **Xiangdong Chen:** Writing – review & editing, Writing – original draft. **Zhiqiang Hu:** Writing – review & editing, Writing – original draft, Visualization, Supervision, Resources, Methodology, Conceptualization.

## Declaration of competing interest

The authors declare that they have no known competing financial interests or personal relationships that could have appeared to influence the work reported in this paper.
